# PKM2 is the Metabolic Checkpoint for Stemness Features in Head and Neck Cancer

**DOI:** 10.7150/jca.132595

**Published:** 2026-08-10

**Authors:** Wan-Hsuan Sun, Ta-Jung Peng, Hsueh-Jou Fang, Chia-Ling Chang, Yu-Wen Tang, Kuang-Hui Sun

**Affiliations:** 1 Division of Head & Neck Surgery, Department of Otolaryngology, Tri-Service General Hospital and National Defense Medical University, Taipei 114, Taiwan; 2 Department of Biotechnology and Laboratory Science in Medicine, Cancer and Immunology Research Center, National Yang Ming Chiao Tung University, Taipei 112, Taiwan; 3 Division of Oral & Maxillofacial Surgery, Department of Stomatology, Taichung Veterans General Hospital, Taichung 407, Taiwan; 4 Department of Education and Research, Taipei City Hospital, Taipei 112, Taiwan

**Keywords:** PKM2, cancer metabolism, cancer stemness, Wnt/β-catenin, head and neck cancer.

## Abstract

Owing to their self-renewal, differentiation capabilities, and increased chemoresistance, cancer stem cells (CSCs) are associated with poor prognosis and an elevated chance of relapse and metastasis. CSCs survive under therapy-imposed pressure and aggravate tumor malignancy through metabolic reprogramming, leading to treatment failure. The RNA level of pyruvate kinase isoenzyme M2 (*PKM2*) is increased in head and neck cancer (HNC) tissues and is linked to poor prognosis, whereas *PKM2* deletion was found to accelerate tumorigenesis and tumor growth *in vivo*, leading to a paradox between *PKM2* expression and tumorigenesis. To date, the role of PKM2 inhibition in HNC progression remains unclear. In the present study, we found that low PKM2 protein expression was significantly associated with later stages and higher grades of cancer in HNC tissues. Following *PKM2* silencing, HNC cells exhibited significantly increased ATP levels, decreased lactate production, and upregulated mitochondrial respiratory activity. In addition, *PKM2* knockdown promoted clonogenicity, motility, nuclear-activated β-catenin, CSC formation, and *in vivo* tumorigenicity in HNC cells. PKM2 inhibition further augmented stemness and resistance to chemotherapeutic drugs in HNC CSCs. Whole-genome transcriptome analysis of *PKM2*-knockdown CSCs revealed upregulation of differentially expressed genes (DEGs) in the Wnt signaling pathway. The present study concluded that decreased PKM2 is associated with aggressive clinicopathological features. PKM2 inhibition-promoted stemness may be mediated by increased nuclear β-catenin localization, which potentially contributes to tumorigenesis and disease progression in HNC. Our findings suggest that PKM2 modulates HNC stemness by linking metabolic rewiring with oncogenic signaling, highlighting its potential as a molecular target for adjuvant therapy in patients with HNC.

## Introduction

Head and neck cancer (HNC) is one of the top ten cancer types among cancer-associated diseases with poor prognosis, even after therapeutic intervention 1. The lack of clinically evident antecedent premalignant lesions often leads to delayed diagnosis. Consequently, most patients suffer from late-stage HNC, which has poor susceptibility to conventional chemoradiotherapy (CRT) and a high risk of subsequent recurrence. Although the application of epidermal growth factor receptor monoclonal antibody (cetuximab) and immune enhancement therapy (pembrolizumab and nivolumab) has contributed to improved performance 2, the elucidation of the molecular genetic landscape and oncogenic hallmarks is indispensable for mining novel, effective, and least-toxic therapeutic interventions for HNC.

Cancer stem cells (CSCs) self-renew and differentiate into multiple lineages, thereby aggravating tumor formation, metastasis, CRT resistance, and relapse. CSCs were previously regarded as a group of quiescent cells. However, studies have found that this population is distinct and metabolically hyperactive, and that homeostasis and tropism are intricately reprogrammed 3. Pyruvate kinase (PK) converts phosphoenolpyruvate to pyruvate and effluxes it for lactate fermentation or the tricarboxylic acid (TCA) cycle. Pyruvate kinase has four isozymes with distinct tissue distributions: PKL, PKR, PKM1, and PKM2. *PKM1* and *PKM2* are alternatively spliced from *PKM*, and include exons 9 and 10, respectively. PKM2 has two structural isoforms, homotetramers and homodimers. The PKM2 tetramer harbors higher catalytic activity to fulfill metabolic demands, whereas the PKM2 dimer serves as a transcriptional co-regulator by nuclear translocation 4.

PKM2 has been reported as a tumor promoter and is involved in many malignant processes, including cancer growth, apoptosis, metastasis, drug resistance, and tumor initiation, by regulating metabolism and signal transduction 4. Clinically, high PKM2 levels are correlated with poor prognosis in lung cancer, ovarian cancer, and multiple myeloma 5-7. In HNC, increased RNA level of *PKM2* is associated with worse outcomes 8. PKM2 protein level is positively associated with advanced clinical stage and poorer survival in oral squamous cell carcinoma (OSCC) and head and neck squamous cell carcinoma (HNSCC) 9,10. From a subcellular perspective, nuclear PKM2 mediates the EGF-promoted cell proliferation and motility in nasopharyngeal carcinoma 11 and drives epithelial-mesenchymal transition (EMT) through proteasome-mediated TGIF2 repression in OSCC 12. Under glucose restriction, PKM2 nuclear translocation sustains pancreatic CSCs and expression of stemness genes 13. Clinically, elevated nuclear PKM2 expression strongly predicts poor prognosis in patients with esophageal squamous cell carcinoma 14. However, the germline loss of *PKM2* promotes hepatocellular carcinoma 15. Deletion of *PKM2* in a mouse model of breast cancer caused by the absence of *Brca1* hastens tumor growth 16. This evidence has led to a paradox between PKM2 expression and tumorigenesis. However, the relationship between PKM2 inhibition and HNC progression also remains unclear.

Here, we demonstrate that PKM2 down-regulation was associated with aggressive clinicopathological features and disease progression in HNC. *PKM2* silencing induced nuclear PKM2/β-catenin co-localization and metabolic rewiring toward mitochondrial respiration, suggesting that PKM2 integrates metabolic and oncogenic signaling to enhance CSC formation, chemoresistance, and *in vivo* tumorigenicity.

## Materials and Methods

### Immunohistochemistry (IHC)

Tissue microarrays were purchased from US Biomax, Inc. (HN802). The disease features and clinical information of the patients with HNC are presented in Table S1. Briefly, the slides were deparaffinized, followed by antigen retrieval and blocking using a Dual Endogenous Enzyme Block (Dako). Anti-PKM2 antibody (1:800, #4053, Cell Signaling Technology) was hybridized at 4 °C overnight, and processed using the EnVision Dual Link System-HRP (DAB) Kit (Dako). Following hematoxylin counterstaining, digital scoring was performed using an Aperio ImageScope. The final score was calculated by adding the percentage of stained areas (0, 1, 2, 3, and 4) and intensity (0, 1, 2, and 3) of the stained cells.

### Cell culture, cell growth, and clonogenic assay

The SAS cell line was derived from a poorly differentiated human squamous cell carcinoma of the tongue and grown in DMEM supplemented with 10% fetal bovine serum (FBS) (Gibco). All experiments were performed using mycoplasma-free cells. Cells (2500/100 μL) were seeded into a 96-well plate, and the MTS assay (Promega) was used to assess cell growth after 24, 48, and 72 h. Cells (50/3 mL) were seeded in a 6-well plate and cultured for 9 days. Colonies were fixed in 100% methanol, stained with 3% crystal violet, and counted to assess clonogenicity.

### *PKM2* knockdown

Short-hairpin RNA (shRNA) oligonucleotides against *PKM2* were synthesized (Mission Biotech) and cloned into the pLKO.1-puro plasmid (Academia Sinica, Taiwan). The target sequences 13 were as follows: sense oligonucleotide: 5'-CCGGCCATAATCGTCCTCACCAACTCGAGTTGGTGAGGACGATTATGGTTTTT-3'; antisense oligonucleotide: 5'-AATTAAAAACCATAATCGTCCTCACCAACTCGAGTTGGTGAGGACGATTATGG-3'. The pCMVΔR8.91, pMD.G, and pLKO.1-puro plasmids were co-transfected into HEK293T cells by TransIT-LT1 reagent (Mirus Bio) to package lentivirus particles as per the instructions from Academia Sinica (Taiwan). HNC cells were transduced with either shLuc or shPKM2 lentivirus (multiplicity of infection (MOI) = 1) in the presence of 8 μg/mL of protamine sulfate (Merck Millipore). Clones resistant to puromycin (1 μg/mL, Sigma-Aldrich) were grown for 14 days to generate a stable population.

### Real-time quantitative polymerase chain reaction (RT-qPCR)

Cells (1 × 10^6^/3 mL) were placed in a 6 cm dish and incubated overnight. RNA was harvested using TRIzol Reagent (Invitrogen), converted to cDNA using the Maximal First Strand cDNA Synthesis Kit (Thermo Scientific), and analyzed using Fast SYBR Green Master Mix with a StepOnePlus real-time PCR system (Applied Biosystems). The primer sequences are listed in Table S2.

### Nuclear/cytoplasm fractionation and western blot

Cells (4 × 10^5^/3 mL) were seeded in a 6 cm dish for 48 h. Cell lysates were prepared using RIPA lysis buffer containing a protease inhibitor cocktail (Thermo Scientific). Cell lysates were fractionated into cytoplasmic (Cyto) and nuclear fractions (Nuc) using column-based extraction kits (Invent Biotechnologies), separated by 10% sodium dodecyl sulfate-polyacrylamide gel electrophoresis, and transferred onto polyvinylidene fluoride membranes. Protein expression levels were assayed using primary antibodies against PKM1, PKM2, activated β-catenin, Sox2, Nanog, histone H3 (Cell Signaling Technology), and β-actin (Sigma-Aldrich), and then visualized by horseradish peroxidase-conjugated secondary antibodies (Jackson ImmunoResearch) coupled with a Pierce ECL substrate (Thermo Scientific).

### Lactate and ATP production

A total of 1 × 10^4^ cells/200 μL were placed in a 96-well plate for 24 h and refreshed with serum-free medium (100 μL) for another 24-h incubation. The lactate levels in the supernatant were determined using an L-Lactate Assay kit (Abcam).

Parental cells (4 × 10^5^/3 mL) were seeded in a 6 cm dish for 48 h. Parental or spheroid cells (4 × 10^4^) were collected and resuspended in 200 μL of distilled water. After three freeze-thaw cycles, ATP production was determined using an ATP Determination Kit (Invitrogen).

### Measurement of oxygen consumption rate (OCR) and extracellular acidification rate (ECAR) by Seahorse XF24 Analyzer

Cells (3 × 10^4^/250 μL) were seeded in a XF24 cell culture microplate for 24 h and refreshed with XF Base Medium Minimal DMEM (Agilent) with supplements (for OCR: 10 mM glucose, 2 mM glutamine, 1 mM sodium pyruvate; for ECAR: 2 mM glutamine). Cells were treated by sequential injection of the following compounds: for OCR: 15 μM oligomycin, 2.5 μM carbonyl cyanide-4-(trifluoromethoxy)phenylhydrazone, and 5 μM rotenone/antimycin; for ECAR: 100 μM glucose, 10 μM oligomycin, and 500 mM 2-deoxy-D-glucose. OCR and ECAR measurements were performed using a Seahorse XF24 Analyzer (Agilent).

### Transwell migration and invasion assay

For migration, SAS cells (3 × 10^4^/100 μL serum-free DMEM) were placed in a Transwell insert (8-μm pore, Corning), and 10% FBS-containing DMEM (800 μL) was added to the bottom chamber and incubated for 30 h.

For invasion, SAS cells (3 × 10^4^ cells/500 μL serum-free DMEM) were seeded in a Matrigel Invasion insert (Corning), and the lower chamber was filled with 10% FBS-containing DMEM (750 μL) for 48 h of incubation. Liu's stain was used to visualize the migrated and invaded cells.

### Sphere formation

SAS shLuc and shPKM2 cells (2.5 × 10^2^ cells/100 μL) were seeded in 96-well ultra-low attachment plates with serum-free DMEM/F12 medium supplemented with 20 ng/mL human epidermal growth factor (EGF), 20 ng/mL human basic fibroblast growth factor (bFGF), and 1% N2 supplement (defined CSC medium; Gibco). The number of spheres was counted after 12 days of incubation. For iCRT-3 (HY-103705; MedChemExpress) treatment, the cells were cultured with the indicated concentrations of iCRT-3; 0.1% dimethyl sulfoxide (DMSO) served as the vehicle control. The half-maximal inhibitory concentration (IC_50_) value was determined using nonlinear regression analysis.

### Chemodrug sensitivity assay of PKM2 knockdown in primary CSCs

SAS cells (1 × 10^4^/2 mL) were cultured in defined CSC medium for 12 days to form primary spheres (1S) and then separated into single cells using 1X Accumax™. Primary spheroid single cells were knocked down by lentiviral shPKM2 infection (MOI: 20) for 3 h. Luciferase shRNA lentivirus (shLuc) was used as a control. shLuc and shPKM2 1S spheroids were cultured in defined CSC medium for 12 days to form secondary spheres (2S).

The secondary spheroid single cells (2 × 10^3^/100 μL) were seeded with varying concentrations of chemodrugs. Cell viability was determined using the CellTiter-Glo Luminescent Cell Viability Assay (Promega) after 48 h of incubation. Cisplatin (P4394), gemcitabine (G6423), and etoposide (E1383) were purchased from Sigma-Aldrich.

### Whole transcriptome analysis

RNA sequencing was performed by Welgene Biotech, Taiwan. In brief, total RNA from pooled biological replicates of shLuc 2S and shPKM2 2S cells was extracted using TRIzol reagent (Thermo Fisher Scientific). RNA was qualified by using a Bioanalyzer 2100 (Agilent Technologies, USA) with an RNA 6000 LabChip kit (Agilent Technologies, USA). A SureSelect XT HS2 mRNA Library Preparation Kit (Agilent Technologies) was used for library construction, and AMPure XP beads (Beckman Coulter) were used for size selection. Sequencing was performed using Illumina sequencing-by-synthesis (SBS) technology, generating 150-bp paired-end reads. Sequencing data (FASTQ reads) were processed using Welgene Biotech's in-house pipeline based on Illumina's base-calling program bcl2fastq v2.20. Transcript assembly and abundance estimation were performed using the StringTie software (v2.1.4). Differential expression analysis was performed using DESeq2 (v1.39.0) with genome bias detection/correction using Welgene Biotech's in-house pipeline. *P-*values was calculated by DESeq with non-grouped samples used in blind mode. Differentially expressed genes (DEGs) were defined as transcripts per million (TPM) > 0.5 and an absolute fold change (|FC|) > 2. For pathway analysis, gene set enrichment analysis (GSEA, software version 4.4.2) was performed in the pre-ranked mode. The gene list was ranked using the following formula: sign(log_2_(FC)) × log(*p*-value). The molecular signatures databases (MSigDB) H (H gene sets), C5 (Gene Ontology: BP gene sets), and C2 (curated gene sets, Kyoto Encyclopedia of Genes and Genomes (KEGG medicus)) collections were used. Gene sets with a false discovery rate (FDR) < 0.25 were considered significantly enriched.

### Dual-luciferase reporter assay

SAS tumorspheres were dissociated into single cells after 12 days of culture. Cells (4×10^5^) were seeded in 6-well plates in sphere medium and co-transfected with the TCF/LEF-driven firefly luciferase construct (Fluc) and a constitutively expressing *Renilla* luciferase construct (Rluc; CCS-018L, QIAGEN). After 48 h of incubation, firefly and *Renilla* luciferase activities were measured using the Twinlite™ Glow Reporter Gene Assay System (Revvity). The Wnt/β-catenin transcriptional activity is expressed as the Fluc/Rluc ratio and normalized to the shLuc control group.

### Subcutaneous xenograft tumor model

NOD/SCID mice (NOD.Cg-*Prkdc^scid^*/JNarl; 6-week-old, male) were obtained from BioLASCO and raised at the National Yang Ming Chiao Tung University Animal Center according to Institutional Animal Care and Use Committee regulations. The SAS parental cells were resuspended in serum-free DMEM, and 1×10^4^ or 1×10^5^ cells in a total volume of 100 μL were subcutaneously injected into the dorsal flanks of three mice. Tumors were measured using calipers at the indicated time points and photographed after excision.

### Statistical analysis

All statistical analyses were conducted using the SPSS software (IBM Corp., Armonk, NY, USA). All experiments were performed with at least three independent biological replicates, and representative data or images are shown. The quantitative data are presented as the mean ± standard deviation (SD). The statistical significance of the data from the cell-based assays was examined using a two-tailed Student's *t*-test. The two-tailed Pearson's chi-square test was conducted to identify the association between PKM2 protein expression and clinicopathological characteristics. The Mann-Whitney U test was performed to examine the variance in PKM2 protein expression between different stages and grades of cancer.

## Results

### Low PKM2 expression is associated with advanced stages and higher grades of cancer in HNC tissues

**Figure 1:**
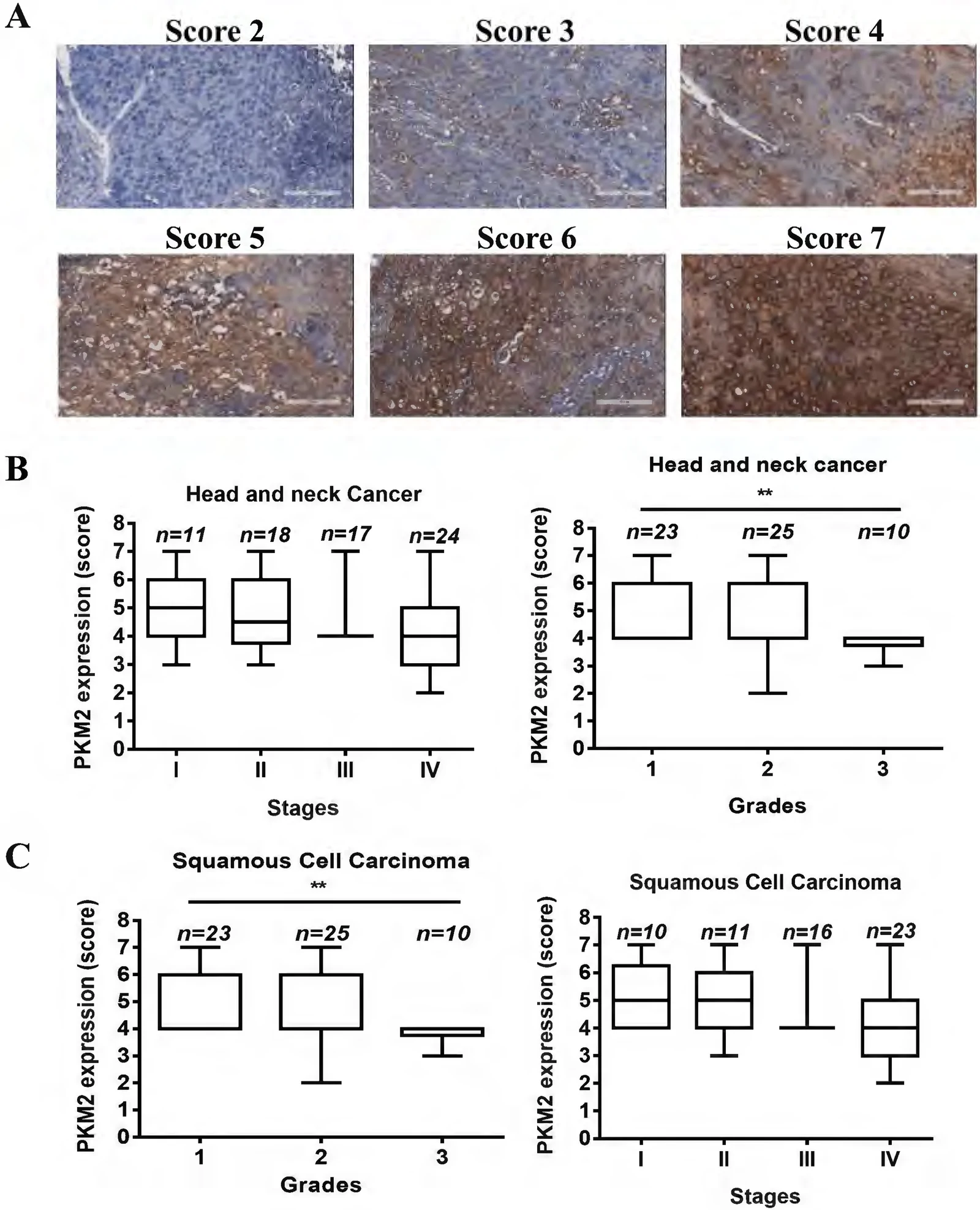
** Lower PKM2 expression is associated with advanced HNC tumor grades. (A)** Representative immunohistochemical images of HNC tissues with different PKM2 expression scores. Scale bar, 100 μm. **(B, C)** Association of PKM2 expression levels with clinical stages and pathological grades in the overall HNC cohort **(B)** and the squamous cell carcinoma sub-cohort **(C).** The Mann-Whitney U test was performed to examine the variance in PKM2 protein expression between different stages and grades of cancer. **p < 0.01

The HN802 tissue microarray was selected for its comprehensive representation of HNC cases across a diverse spectrum of clinical stages (I-IV) and pathological grades (1-3), which is essential for robust statistical evaluation of the association between PKM2 expression and disease progression. We performed IHC staining of HNC tissue microarrays to explore the clinical relevance of PKM2 protein expression **(Fig. 1A)**. The tissues were scored from 2 to 7 as follows: 2 (*n* = 1), 3 (*n* = 11), 4 (*n* = 32), 5 (*n* = 11), 6 (*n* = 8), and 7 (*n* = 7). The median expression level is the most frequently used criterion for expression-based patient stratification. Therefore, we separated the cohort into a low expression group (scores ≤ 4, *n* = 44) and a high expression group (scores ≥ 5, *n* = 26) **(Table 1)**. Although PKM2 expression levels were not associated with sex, age, T stage, node involvement (N), or metastasis (M), a higher percentage of PKM2 low levels was significantly associated with advanced stages (31/41, 75.6%) and high grades (10/10, 100%) in patients with HNC **(Table 1)**. PKM2 levels were significantly higher in grade 1 than in grade 3 in all HNC and in the squamous cell carcinoma cohort **(Fig. 1B, 1C)**. These results revealed that PKM2 expression negatively correlated with HNC progression.

### Increased cytoplasmic PKM1, nuclear PKM2, and nuclear activated β-catenin in *PKM2*-silenced HNC cells

**Figure 2:**
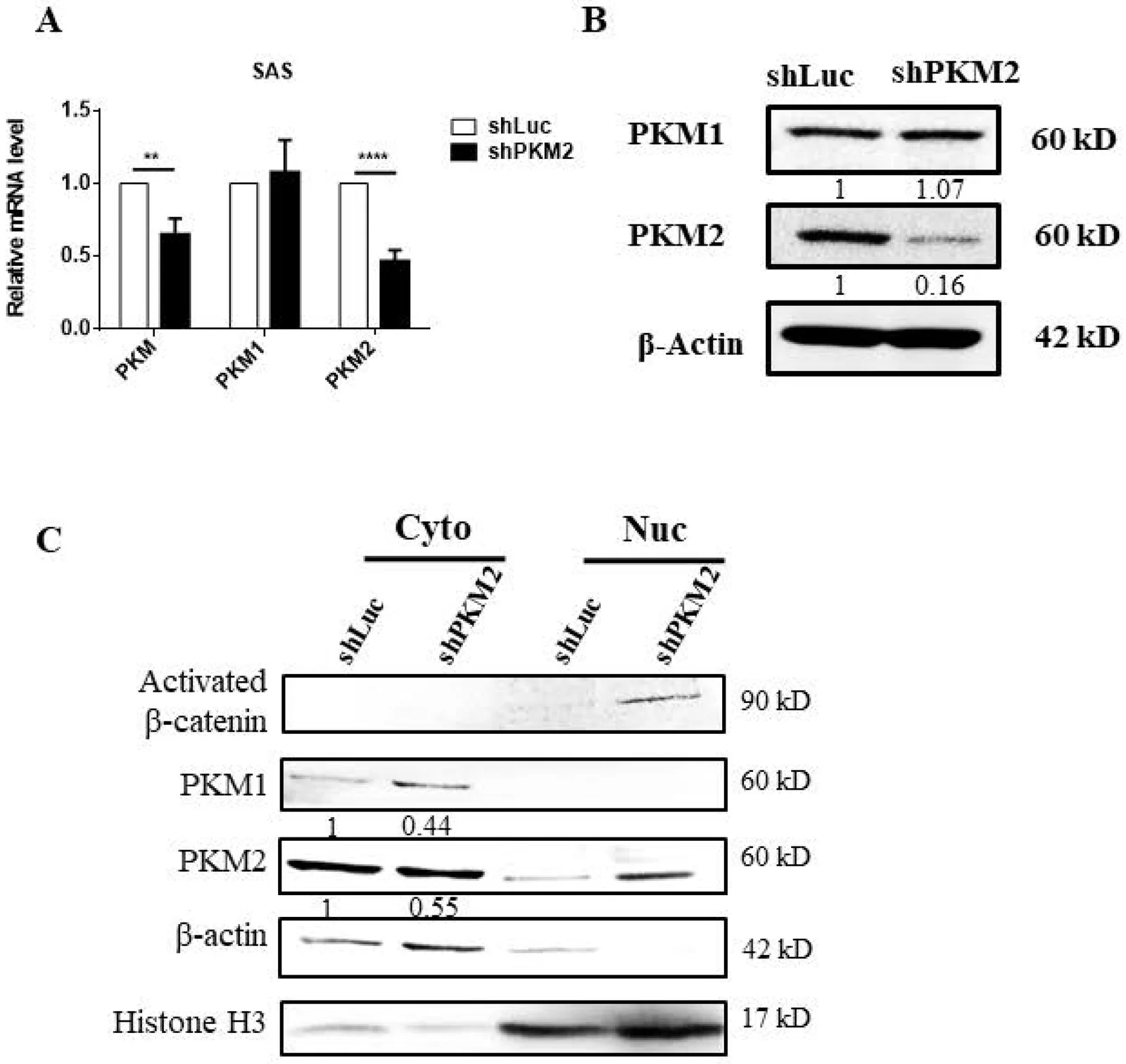
** Nuclear PKM2 and activated β-catenin levels are increased in *PKM2* knockdown cells. (A)** Relative mRNA expression levels of *PKM*, *PKM1*, and *PKM2* in SAS shLuc and shPKM2 parental cells determined by RT-qPCR. **(B, C)** Western blot analysis of the indicated proteins in whole-cell lysates **(B)** and subcellular fractions (Cyto: cytoplasmic; Nuc: nuclear) **(C)** of SAS shLuc and shPKM2 parental cells. *p < 0.05, **p < 0.01, ****p < 0.0001.

The SAS cell line, a well-characterized human papilloma virus-negative cell line harboring a *TP53* nonsense mutation (p.E336Ter), is derived from a poorly differentiated human OSCC of the tongue. The oral cavity is the most prevalent and lethal subtype of HNC 17. This specific virologic and genomic profile makes it the most clinically challenging and therapy-resistant subtype of HNC 18, thereby providing a highly relevant model to investigate the factors driving disease progression. To investigate the role of PKM2 in HNC progression, an shRNA lentivirus system was used to knockdown *PKM2* (shPKM2) in SAS cells. PKM2 expression was decreased by approximately 50% at both the RNA **(Fig. 2A)** and protein levels** (Fig. 2B)** in shPKM2 SAS cells. Because PKM2 can translocate into the cell nucleus, we fractionated the cell lysates into cytoplasmic and nuclear fractions and performed western blotting to determine the cellular localization of the proteins. PKM2 was found in both the cytoplasm and the nucleus, whereas PKM1 was found only in the cytoplasm **(Fig. 2C)**. Furthermore, *PKM2* silencing upregulated cytoplasmic PKM1, nuclear PKM2, and nuclear activated β-catenin in SAS cells.

### Decreased lactate, increased ATP production, and upregulated mitochondrial respiratory activity in *PKM2*-silenced HNC cells

**Figure 3:**
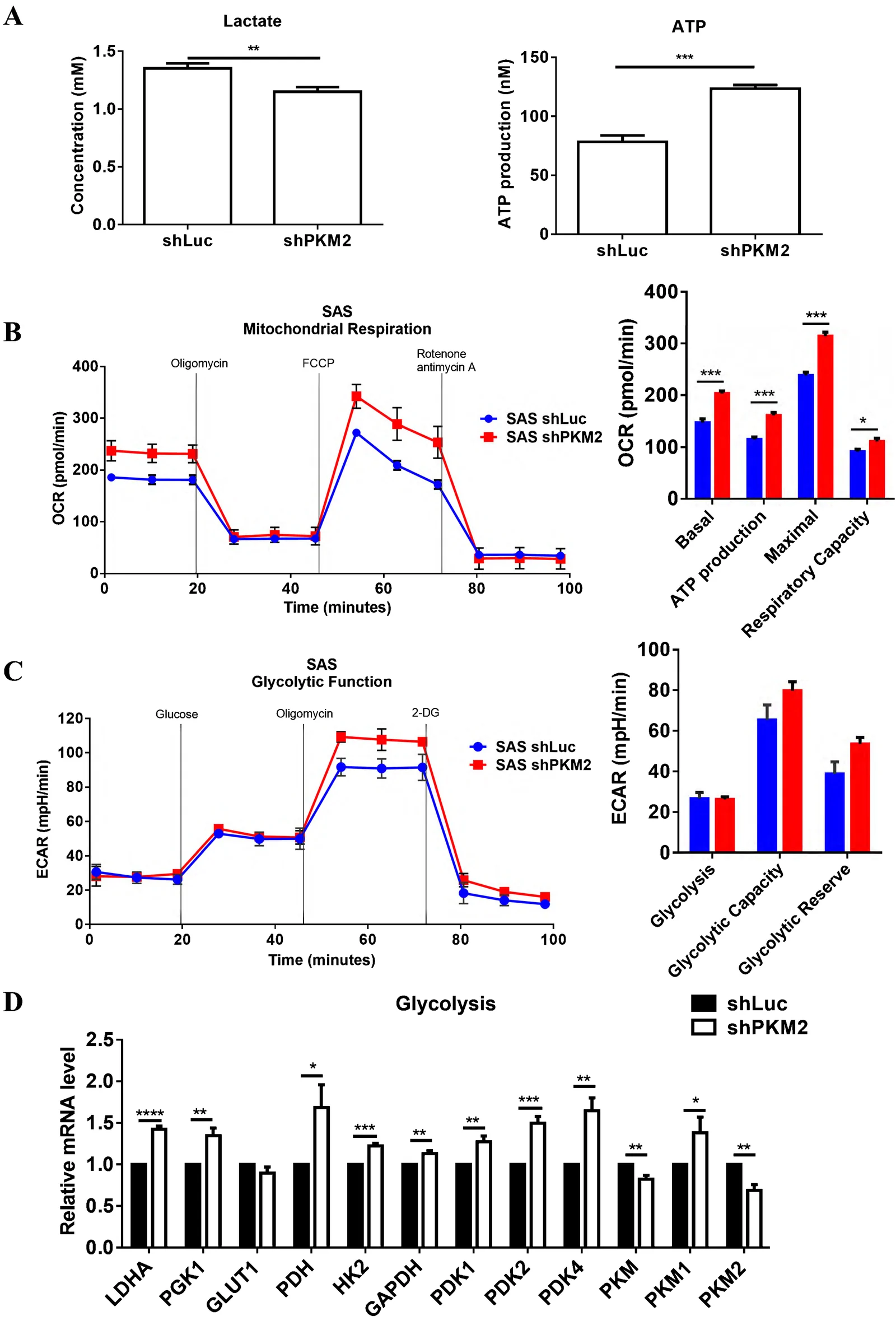
***PKM2* knockdown increases mitochondrial respiratory capacity. (A)** Relative extracellular lactate production and intracellular ATP levels in SAS shLuc and shPKM2 parental cells. **(B, C)** Real-time oxygen consumption rate (OCR) **(B)** and extracellular acidification rate (ECAR) **(C)** measured by Seahorse XF analysis. **(D)** Relative mRNA expression of metabolism-related genes in SAS shLuc and shPKM2 parental cells determined by RT-qPCR. *p < 0.05, **p < 0.01, ***p < 0.001.

We analyzed the effects of *PKM2* knockdown on cellular metabolism. Increased ATP levels and decreased lactate production were observed in the *PKM2*-silenced SAS cells **(Fig. 3A)**. Through real-time live-cell assessment using a Seahorse XF24 analyzer, we measured the OCR, which represents mitochondrial oxidative phosphorylation, and the ECAR, which measures glycolysis. *PKM2* silencing induced a significant increase in OCR profiles, such as basal respiration, ATP production, maximal respiration, and respiratory capacity in SAS cells **(Fig. 3B)**. However, basal glycolysis in the ECAR profiles remained unaffected, and glycolytic capacity and reserves were slightly increased in shPKM2 SAS cells **(Fig. 3C)**. Moreover, *PKM2* knockdown increased the mRNA expression of *LDHA*, *PGK1*, *PDH*, *HK2*, *PDK*, *GAPDH*, and *PKM1* in SAS cells **(Fig. 3D)**. Therefore, PKM2 inhibition promotes mitochondrial oxidative phosphorylation in SAS cells.

### *PKM2* knockdown promoted clonogenicity, migration, and invasion ability in SAS cells

**Figure 4:**
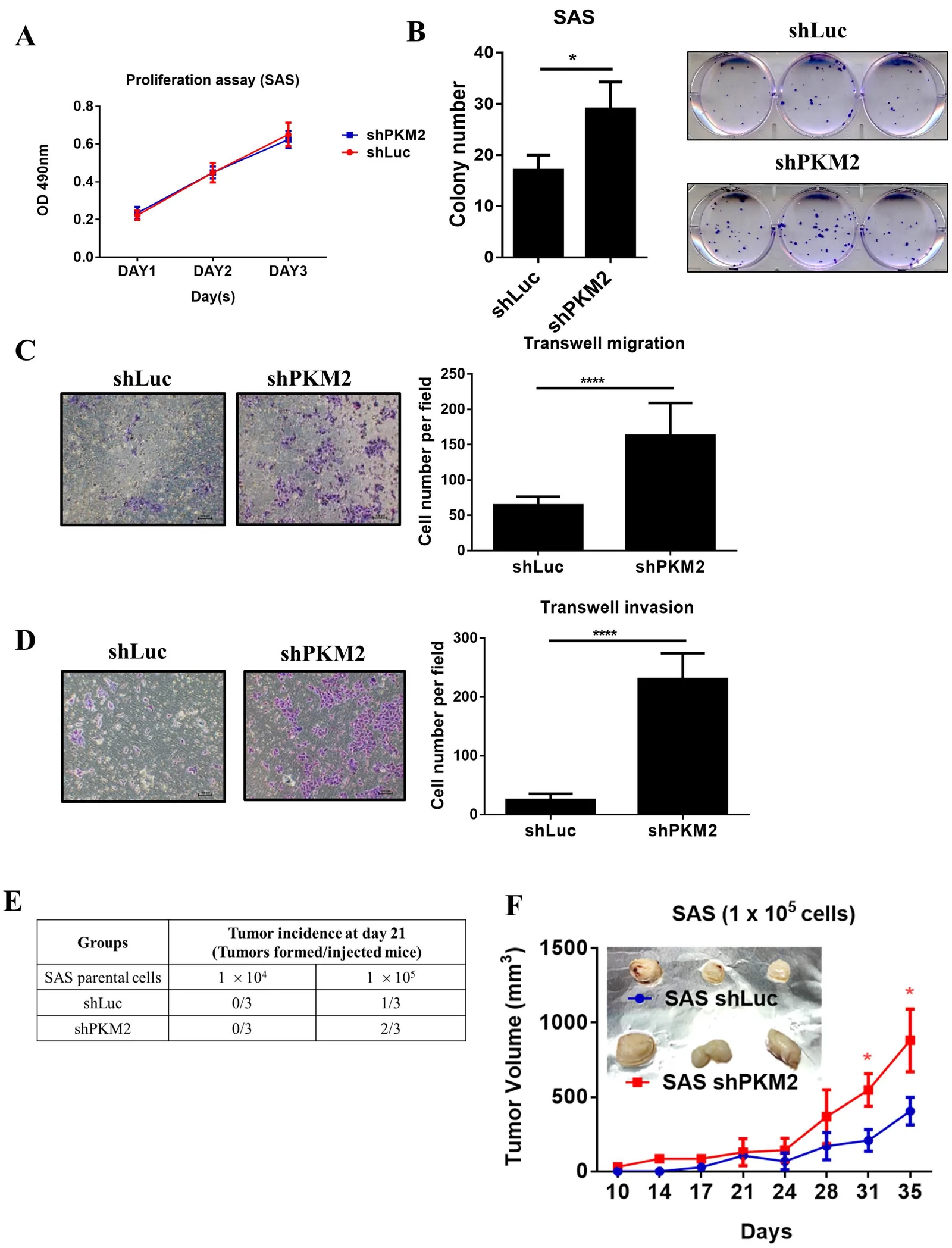
***PKM2* knockdown increases long-term growth and motility of cells**. **(A)** Proliferation of SAS parental cells assessed by MTS assay. **(B)** Clonogenicity of SAS shLuc and shPKM2 cells evaluated by colony formation assay. **(C, D)** SAS parental cell migration **(C)** and invasion **(D)** capabilities determined by Transwell assays. Scale bars, 100 μm. (E, F) shLuc and shPKM2 SAS parental cells were subcutaneously inoculated into NOD/SCID mice. (E) The tumor incidence at day 21. (F) The tumor volume was measured at the indicated time points. The image showed the excised tumor at the experimental endpoint. *p < 0.05, ****p < 0.0001.

We investigated the effect of *PKM2* knockdown on cell growth and motility. *PKM2* silencing did not alter the 3-day cell proliferation **(Fig. 4A)**. However, *PKM2* knockdown significantly promoted the ability of a single cell to grow into a colony (clonogenicity) after a 9-day culture **(Fig. 4B)**. In addition, *PKM2* silencing significantly increased the Transwell migration** (Fig. 4C)** and invasion activity **(Fig. 4D)** of SAS cells. We constructed a subcutaneous xenograft model to investigate the effects of PKM2 inhibition on tumorigenesis *in vivo*. We found that shPKM2 cells showed increased tumor incidence after 21 days and increased tumor volume at the end of the experiment compared to shLuc cells **(Fig. 4E, 4F).**

### PKM2 inhibition augments the sphere-forming capability, stemness, and chemoresistance in CSCs

**Figure 5:**
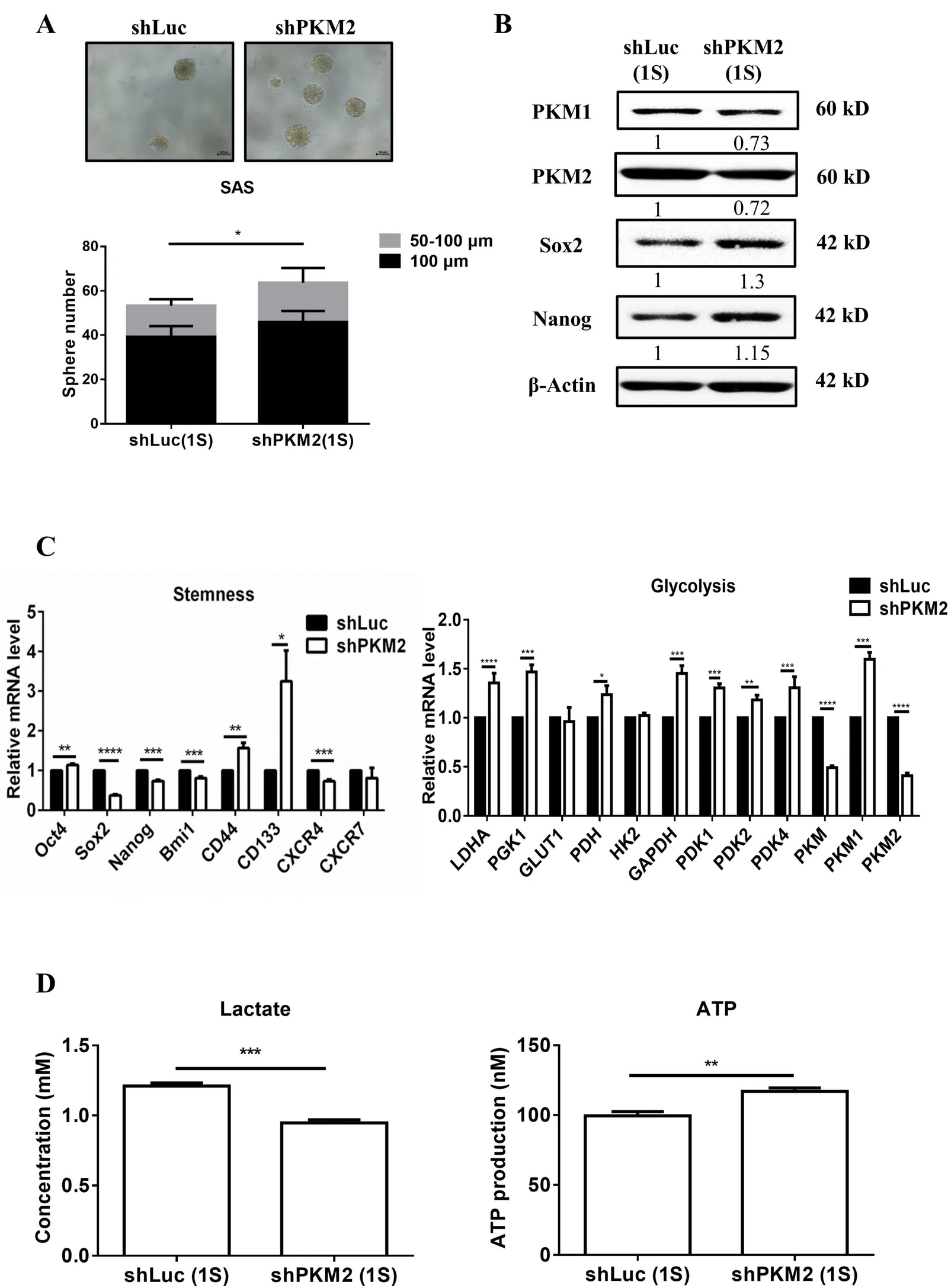
***PKM2* knockdown promotes tumorsphere formation and cancer stemness properties. (A)** Representative images and quantification of primary tumorspheres (1S) formed by SAS shLuc and shPKM2 cells. Scale bar, 100 μm. **(B)** Western blot analysis and corresponding quantification of stemness-related markers, PKM1, and PKM2 in 1S cells. **(C)** Relative mRNA expression of stemness and metabolism genes in SAS 1S cells. **(D)** Relative extracellular lactate production and intracellular ATP levels in 1S cells. *p < 0.05, **p < 0.01, ***p < 0.001.

CSCs have self-renewal and differentiation capabilities and increased chemotherapy resistance. Therefore, we investigated the effects of PKM2 inhibition on the properties of CSCs. *PKM2* silencing significantly augmented sphere formation **(Fig. 5A)**. The stem cell transcription factors Sox2 and Nanog increased in shPKM2 sphere cells after 12-day CSC culture **(Fig. 5B)**. The mRNA levels of stem cell markers (*OCT4*, *CD44*, and *CD133*) and glycolytic genes (*LDHA*, *PGK1*, *PDH*, *PDK*, *GAPDH*, and *PKM1*) were upregulated in PKM2-silenced spheres **(Fig. 5C)**. Furthermore, decreased lactate production and increased ATP production were observed in shPKM2 spheroid cells **(Fig. 5D)**. Thus, PKM2 inhibition increases the sphere-forming capability and stemness of HNC cells.

**Figure 6:**
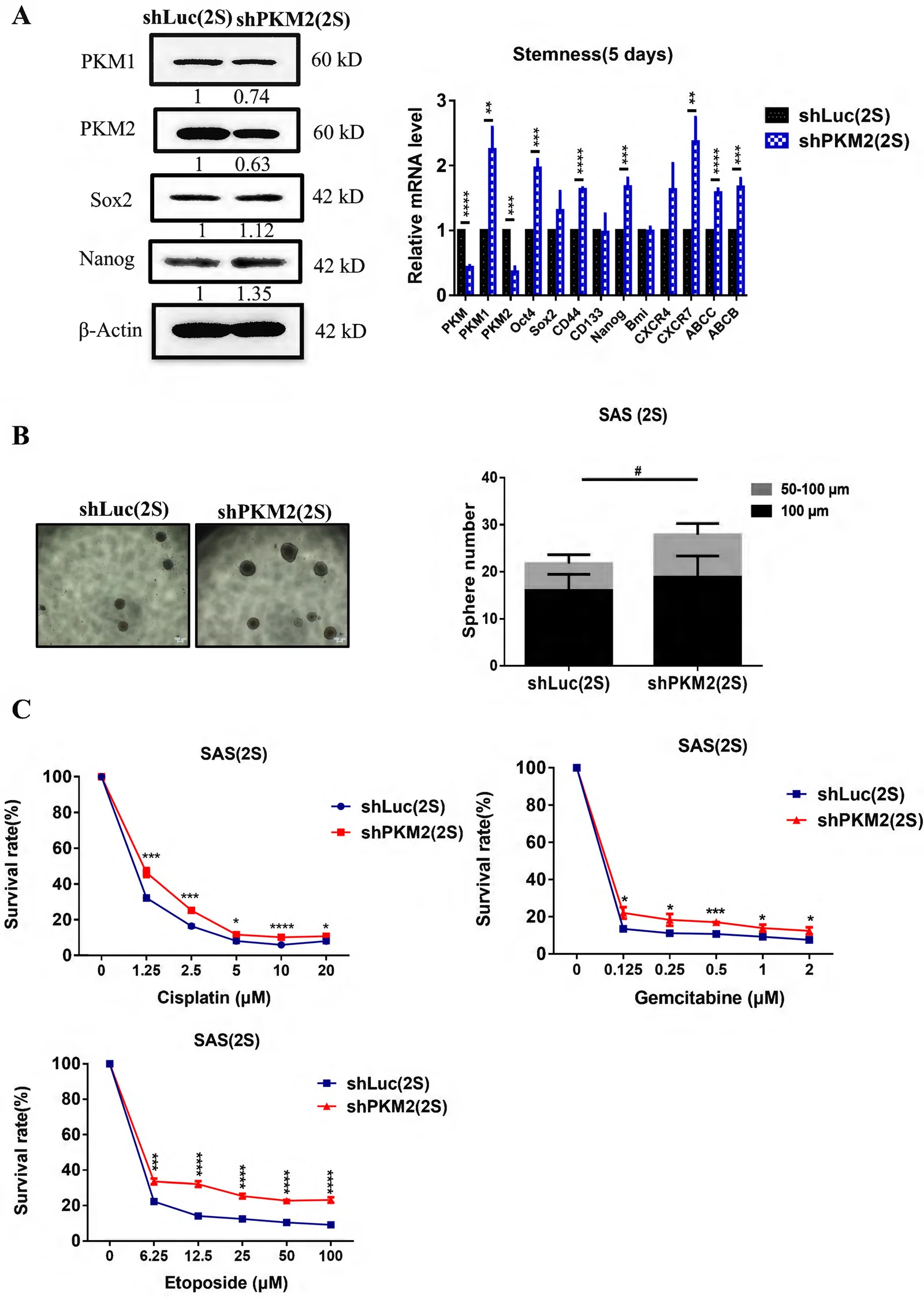
***PKM2* knockdown in cancer stem cells increases motility and chemodrug resistance. (A)** Protein (left panel) and mRNA (right panel) expression levels of stemness markers in SAS secondary spheres (2S). **(B)** Representative images and quantification of 2S formation. Scale bar, 100 μm. **(C)** Cell viability of 2S cells following 48-h treatment with the indicated concentrations of chemodrugs. *p < 0.05, **p < 0.01, ***p < 0.001, ****p < 0.0001.

*PKM2* was knocked down in CSC-enriched SAS 1S to investigate its role in maintaining the stemness of HNC CSCs. SAS 1S cells were infected with shLuc or shPKM2 lentiviruses and cultured to form secondary spheres (2S). After verifying the inhibition of PKM2 at the gene and protein levels, we observed increased Sox2 and Nanog protein levels** (Fig. 6A**, left panel**)** in shPKM2 2S cells. Additionally, the mRNA levels of *PKM1*, stemness genes (*OCT4*, *SOX2*, *NANOG*, *CD44*, *CXCR4*, and *CXCR7*), and multiple drug resistance genes (*ABCC* and *ABCB*) were upregulated **(Fig. 6A**, right panel**)**. Moreover, the suppression of PKM2 in SAS 1S cells promoted sphere formation, with larger size and greater numbers **(Fig. 6B)** and chemotherapeutic resistance (cisplatin, gemcitabine, etoposide; **Fig. 6C**) in CSC-enriched SAS cells. Thus, PKM2 downregulation enhances the self-renewal capacity of SAS CSCs.

### DEGs of *PKM2* knockdown in HNC CSCs showed upregulated Wnt signaling

**Figure 7:**
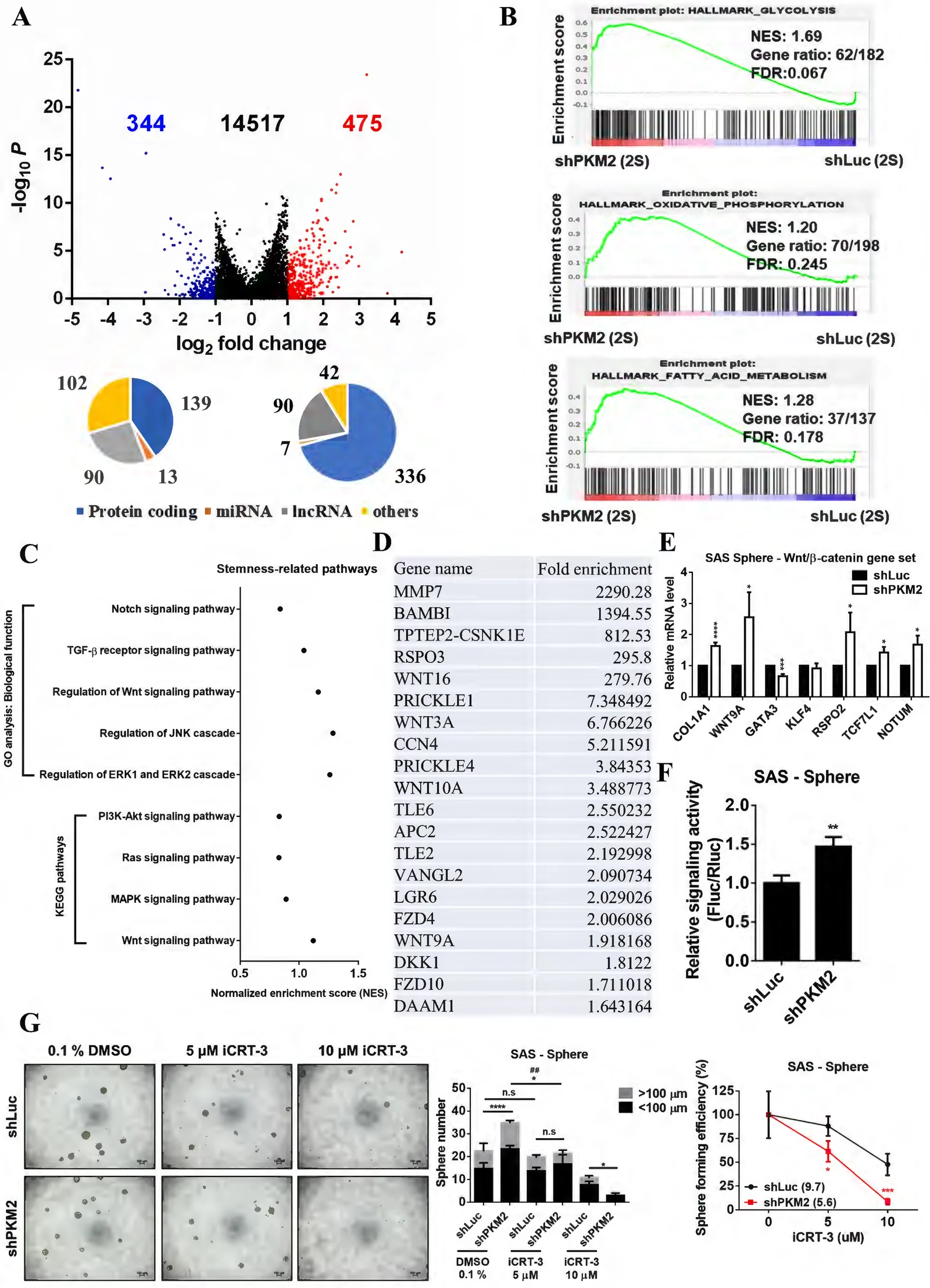
** Differentially expressed genes (DEGs) determined by RNA sequencing of *PKM2* knockdown in cancer stem cells. (A)** Volcano plot of DEGs in shPKM2 versus shLuc 2S cells. Significant DEGs (TPM > 0.5 and |fold change| > 2) are highlighted in red (upregulated) and blue (downregulated). **(B, C)** Normalized enrichment scores (NES) of metabolism-related **(B)** and stemness-related **(C)** pathways identified by Gene Set Enrichment Analysis (GSEA). **(D)** List of core enrichment genes within the Wnt signaling pathway (hsa04310). **(E)** mRNA expression levels of Wnt/β-catenin in SAS 1S. **(F)** Wnt/β-catenin signaling activity in SAS 1S was determined by TCF/LEF reporter assay. **(G)** Representative images and quantification of primary tumorspheres (1S) formed by SAS shLuc and shPKM2 cells with the indicated concentrations of iCRT-3 or 0.1% DMSO (vehicle control). Scale bar, 100 μm. *: comparison of smaller tumorspheres; #: comparison of larger tumorspheres. Sphere-forming efficiency is expressed as a percentage relative to the corresponding vehicle group.

To investigate the molecular alterations underlying *PKM2* silencing in CSCs, a whole-genome transcriptome analysis was performed using shPKM2 and shLuc 2S cells. Analysis of DEGs using a cutoff change of over 2-fold identified 819 genes, of which 475 were upregulated and 344 were downregulated **(Fig. 7A)**. DEGs included protein-coding RNA, microRNAs, and long non-coding RNA. GSEA was performed for GO terms in metabolic pathways **(Fig. 7B)***. GSEA recommends an FDR threshold of 0.25* in the setting of exploratory discovery. These results revealed a set of enriched processes related to glycolysis, oxidative phosphorylation, and fatty acid metabolism. In addition, stemness-related pathways examined by GO and KEGG enrichment analysis indicated that the TGF-β, Wnt, JNK, and ERK signaling pathways were upregulated (normalized enrichment score, NES >1) in shPKM2 CSC 2S cells** (Fig. 7C)**. The upregulated Wnt signaling pathway was found in both the GO and KEGG enrichment analyses** (Fig. 7C)**; and the core enrichment genes in the Wnt signaling pathway are listed **(Fig. 7D)**. The Wnt family (*WNT16*, *WNT3A*, *WNT10A*, *WNT9A*), frizzled receptors (*FZD4*, *FZD10*), and *RSPO3* (activator of the Wnt/β-catenin signaling) were increased prominently. The upregulation of *WNT9A* and *RSPO2* in shPKM2 CSCs was confirmed using RT-qPCR **(Fig. 7E).** A firefly/*Renilla* dual-luciferase reporter assay with TCF/LEF responsive elements confirmed the enhanced transcriptional activity of Wnt/β-catenin signaling in *PKM2*-silenced CSCs** (Fig. 7F).** To examine the dependency on β-catenin activity in *PKM2* silencing-promoted cancer stemness, we performed sphere formation assay with iCRT-3, an inhibitor of Wnt/β-catenin-responsive transcription. Five μM iCRT-3 attenuated the sphere number that was increased by *PKM2* silencing. Ten μM iCRT-3 exhibited a higher inhibitory effect on the sphere-forming capacity of *PKM2*-silenced cells compared with shLuc control cells. The decreased IC_50_ value to iCRT-3 treatment indicated that shPKM2 tumorspheres were more susceptible to Wnt/β-catenin signaling inhibition **(Fig. 7G)**. Increased nuclear β-catenin was observed in shPKM2 parental cells **(Fig. 2C)**. Taken together, the findings indicate that increased Wnt signaling activators and nuclear β-catenin may potentially mediate the shPKM2-promoted HNC cell stemness.

## Discussion

CSCs have different metabolic features and plasticity based on their oncogenic background, tissue origin, and tumor microenvironment. The metabolic plasticity of CSCs promotes self-renewal, CRT resistance, immune evasion, metastasis, and recurrence 3. Co-targeting of CSCs with metabolic inhibitors and systemic therapeutic drugs is more effective in overcoming CRT resistance and prolonging survival in patients with cancer 3. Thus, exploring the molecular mechanisms underlying the metabolic plasticity of CSCs may reveal novel metabolic targets for combination therapies in CSC-driven tumor advancement.

The allosteric switch between the cytoplasmic tetramer and nuclear dimer of PKM2 is crucial for cancer cells to protect against environmental stress by coordinating metabolic and signaling activities. The low glycolytic activity of dimeric PKM2 shifts metabolic tropism from the TCA cycle to lactate production and upregulates nucleotide biosynthesis via the pentose phosphate pathway to promote tumor growth. In addition, it functions as a transcriptional coactivator in the nucleus to regulate cancer cell growth, metastasis, stemness, and drug resistance 4. After *PKM2* silencing, we observed metabolic rewiring with decreased lactate and increased ATP production in *PKM2*-silenced SAS parental cells **(Fig. 3A)** and CSCs **(Fig. 5D)** led by decreased cytoplasmic distribution** (Fig. 2C)**. Moreover, *PKM2* knockdown upregulated fatty acid metabolism in CSCs** (Fig. 7B)**, compensating for the glycolytic deficit and promoting CSC proliferation.

*PKM2* silencing and inhibition of PKM2 nuclear translocation attenuates EGF-enhanced nasopharyngeal carcinoma cell motility 11. PKM2 inhibition significantly reduces lung cancer growth in mice 19. In addition, siPKM2 suppresses the proliferation and migration of OSCC and HNSCC cells 20,21. However, Tanaka* et al.* demonstrated that siPKM2 only inhibited OSCC cell motility under EMT induction by TGF-β1 12. In the present study, we found that *PKM2* knockdown did not affect 3-day cell proliferation, but promoted the clonogenicity and motility of SAS cells **(Fig. 4)**. Therefore, the experimental approach and conditions may have affected the effects of PKM2 inhibition *in vitro.* Oncogenic background, tissue origin, and context-specific metabolic tropism may determine the function of PKM2.

PKM2-promoted cancer stemness through the Wnt/β-catenin pathway was first found in breast tumors 22. Yang *et al.* demonstrated that nuclear PKM2 is critical for β-catenin-driven transcriptional activity and cancer progression by forming a physical interaction complex 23. Glucose restriction-induced nuclear co-translocation of PKM2/AMPK promotes the binding of PKM2 to Oct4, transactivates stemness-related genes, and induces *in vivo* metastasis 13. Endogenous p-Tyr105 PKM2 levels correlate with CD44, a stemness marker, in HNC cells 24. ErbB2-mediated p-Tyr105 induces PKM2 dimerization and nuclear translocation, and promotes cancer stem-like cells by increasing YAP nuclear translocation 25. Shikonin, a PKM2 phosphorylation inhibitor, exhibits antitumor efficacy by impeding the stemness population during combination treatment 20,21,26,27. Suzuki *et al*. found that PKM2 targeted by shRNA or an inhibitor (2825-0090) downregulated cytoplasmic PKM2 but upregulated nuclear PKM2 in lung cancer cells 19. Our study demonstrated the increased nuclear localization of PKM2 and β-catenin after *PKM2* silencing **(Fig. 2C)**, which may contribute to the Wnt/β-catenin signaling activation and be associated with the enhanced cancer stemness **(Fig. 7E-G)**.

The PKM2 inhibitor (TT-232) showed promise in a phase II clinical trial for metastatic kidney cancer 28. The nuclear translocation of PKM2 induced by TT-232 was sufficient to induce cell death 29. Paradoxically, *PKM2*-knockout mice develop spontaneous hepatocellular carcinoma and show accelerated onset of spontaneous breast cancer tumorigenesis 15,16. Germline loss of the *PKM2* can induce spontaneous hepatocellular carcinoma and lead to a unique metabolite profile, accompanied by multiple carcinogenic driving events, abnormal fatty acid metabolism, liver inflammation, and increased hepatocyte proliferation 15. *PKM2* deletion results in PKM1 compensation and sustained glucose metabolism in *PKM2-*null spontaneous breast tumors 16. Systemic *PKM2* knockout increases plasma cholesterol levels and promotes allograft breast tumor growth 30. We confirmed that *PKM2* silencing promoted HNC stemness in *PKM2*-knockout SAS cells, which recapitulated the decreased lactate secretion, increased ATP production, enhanced tumorsphere formation, and upregulated the expression of stemness markers in SAS CSCs (data not shown). Our findings suggested that the loss of PKM2's canonical glycolytic function may serve as a metabolic trigger driving stemness features, mirroring *in vivo* studies where PKM2 deficiency accelerated spontaneous tumorigenesis with altered glucose and lipid homeostasis in cancers 15,16,30. Our shRNA model further suggested that an additional layer of the PKM2 inhibition-exacerbated phenotype was associated with increased nuclear translocation of the residual PKM2 pool** (Fig. 2C).** Therefore, broad *in vitro* and *in vivo* models should be further investigated to consider the generalizability of PKM2 inhibition-promoted HNC stemness properties and the long-term clinical use of PKM2 inhibitors, which may induce CSC formation, CRT resistance, and cancer relapse.

Studies have linked high *PKM2* mRNA expression to poor prognosis in various malignancies 5,6,8. Increased PKM2 protein expression is associated with aggressive clinicopathological features and unfavorable prognosis in OSCC and HNSCC 9,10. In the present study, quantification of total PKM2 protein by IHC staining showed that low PKM2 expression was significantly associated with advanced stages and higher grades of HNC **(Fig. 1 and Table 1).** Biologically, high PKM2 expression translates into an abundant protein pool that simultaneously supports the formation of tetramers (cytosolic: glycolysis) and dimers (nuclear: oncogenic transcription), cooperatively driving tumor progression. Post-transcriptional regulation may contribute to the discrepancy between high mRNA and low protein levels (e.g., miR-139-5p) 31. Furthermore, the function of PKM2 depends on its subcellular localization 4, which cannot be distinguished using transcriptomic analysis.

Notably, our *in vitro* data suggest that the discrepancy in reduced total PKM2 protein expression drives a poorly differentiated status by forcing the remaining PKM2 pool to undergo nuclear translocation **(Fig. 2B, 2C).** Accumulated evidence consistently indicates that elevated nuclear PKM2 is driven by specific post-translational modifications, including Y105 phosphorylation 25, S37 phosphorylation 32, K433 acetylation 33, and K62 deacetylation 34. These post-translational modifications serve as promoters of EMT, advanced tumor stage, and poor prognosis across various malignancies 10,12,14. Furthermore, PKM2-inhibitor non-responding tumors demonstrated low cytoplasmic staining and a more than 38% increase in nuclear PKM2 staining 19. Notably, Apostolidi *et al.* found that the expression of S37**-**phosphorylated nucleus-localized PKM2, but not total PKM2, was correlated with decreased overall survival in patients with triple-negative breast cancer 32. This growing evidence indicates that quantification of the nuclear/cytosol ratio, which remains limited in the present study, and the post-translational modification status of PKM2 may be more decisive factors in evaluating clinical outcomes and patient stratification than bulk mRNA or total protein expression. In addition, context-dependent roles and tumor heterogeneity should be carefully considered when interpreting the clinical relevance of PKM2.

**Figure 8:**
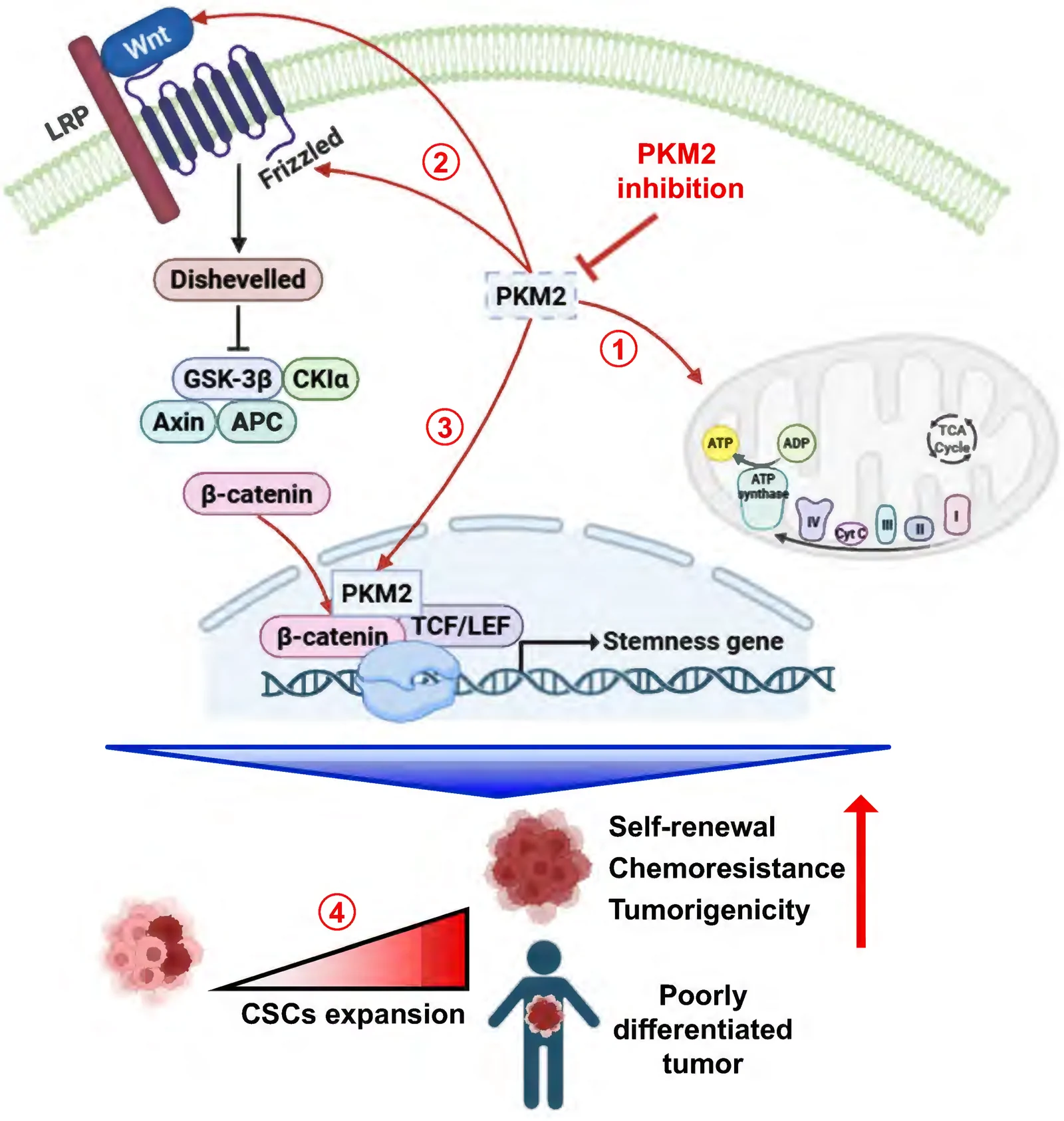
** Graphical abstract.** PKM2 inhibition enhances mitochondrial respiratory activity (1) and upregulates Wnt-FZD expression, thereby driving β-catenin nuclear translocation (2). The increased nuclear PKM2 translocation may form a complex with β-catenin and promote signaling transactivation (3), which consequently augments CSC formation, self-renewal capacity, chemoresistance, tumorigenicity, and initiates poorly differentiated tumors in patients with head and neck cancer (4).

In this study, we demonstrated that *PKM2* silencing mediates metabolic reprogramming, promotes tumorigenesis *in vivo*, and contributes to CSC properties in HNC cells, which may be attributed to the increased PKM2 nuclear localization and Wnt/β-catenin activation. We suggest that nuclear PKM2 is a potential theranostic target for combined treatment of HNC. Inhibitors that block PKM2 post-translational modifications and nuclear translocation may improve therapeutic efficacy. Notably, the potential risk of long-term PKM2 inhibition leading to CSC expansion, therapeutic resistance, and tumor recurrence should be carefully considered** (Fig. 8).**

## Figures and Tables

**Table 1:** Association of PKM2 expression with clinical features of patients with HNC.

	No. of patients with HNC		PKM2^a^	
		Low	High	p-value
Sex				
F	14	7	7	0.266
M	56	37	19	
Stage				
I+II	29	13	16	0.009
III+IV	41	31	10	
Age (years)				
< 55	40	26	14	0.668
≥ 55	30	18	12	
T				
T1+T2	37	20	17	0.107
T3+T4	33	24	9	
N				
N0	51	19	32	0.253
N1+N2	19	7	12	
M				
M0	67	41	26	0.264
M1	2	2	0	
Grade				
1+2	48	26	22	0.007
3	10	10	0	

PKM2^a^: Low score =2-4, High score =5-7. T, primary tumor size; N, lymph node metastasis; M, distant metastasis. HNC, head and neck cancer. Two-tailed Pearson's chi-square test was conducted to identify the association between PKM2 protein expression and clinicopathological characteristics.

## Data Availability

Data is available on request from the authors.
