# Supplementary material for: PKM2 is the Metabolic Checkpoint for Stemness Features in Head and Neck Cancer
Source: J Cancer. 2026 Aug 10;17(8):1504–19. doi: 10.7150/jca.132595 (PMC13505365; doi:10.7150/jca.132595)
Supplement: Supplementary file 1 [file jcav17p1504s1.pdf]

Supplementary Table 1. Clinicopathologic characteristics of patients with head and neck cancer included in this study

Characteristics	No.	Characteristics	No.
Sex		N classification	
Female	14	N0	51
Male	56	N1	12
Stage		N2	7
I	11	M classification	
II	18	M0	68
III	17	M1	2
IV	24	Grade	
Age (years)		Not applicable	12
＜55	37	1	23
≧55	33	2	25
T classification		3	10
T1	13	Anatomic site	
T2	24	Pharynx	5
T3	13	Nasal cavity	8
T4	20	Oral cavity	20
		Larynx	37

Supplementary Table 2. Primer sequences used in RT-qPCR

Genes	Forward primer/Reverse primer
PKM	5'-GTCGAAGCCCCATAGTGAAG-3'<br>5'- GTGAATCAATGTCCAGGCGG -3'
PKM1	5'-TCACTCCACAGACCTCATGG-3'<br>5'- GAAGATGCCACGGTACAGGT -3'
PKM2	5'- AGGC TGCCATCTACCACTTG -3'<br>5'- CACTGCAGCACTTGAAGGAG -3'
PDK1	5'-CACCATGCCAACAGAGGTGTT-3'<br>5'-CCTCATTACCCAGCGTGACA-3'
PDK2	5'-TACGTCCCCTCCCACCTCTA-3'<br>5'-GCCCTCATGGCATTCTTGA-3'
PDK4	5'-CCTTTGGCTGGTTTTGGTTA-3'<br>5'-CCTGCTTGGGATACACCAGT-3'
PDH	5'-GGCGCAAGTTGGTGATCCTA-3'<br>5'-CTCCAGAAAATGCCACTCGAA-3'
LDHA	5'-AGCGGTTGCAATCTGGATTC-3'<br>5'-GGTGAACTCCCAGCCTTTCC-3'
GLUT1	5'-TCACTGTGCTCCTGGTTCTG-3'<br>5'-CCTGTGCTCCTGAGAGATCC-3'
HK2	5'-CAAAGTGACAGTGGGTGTGG-3'<br>5'-GCCAGGTCCTTCACTGTCTC-3'
Nanog	5'-CCCAAAGGCAAACAACCCACTTCT-3'<br>5'-AGCTGGGTGGAAGAGAACACAGTT-3'
Sox2	5'-GCACATGAACGGCTGGAGCAACG-3'<br>5'-TGCTGCGAGTAGGACATGCTGTAGG-3'
Oct4	5'-GTGGAGGAAGCTGACAACAA-3'<br>5'-ATTCTCCAGGTTGCCTCTCA-3'
CD44	5'-CCGCTATGTCCAGAAAGGA-3'<br>5'-CTGTCTGTGCTGTCGGTGAT-3'
CD133	5'-TGGATGCAGAACTTGACAACGT-3'<br>5'-ATACCTGCTACGACAGTCGTGGT-3'
Bmi-1	5'-TGGAGAAGGAATGGTCCACTTC-3'<br>5'-GTGAGGAAACTGTGGATGAGGA-3'
ABCC1	5'-TGCTGCACCAGTACTTCCACAT-3'<br>5'-CCCCAATGACAGCGGTCTT-3'
ABCG2	5'-GCAGCTCTTCGGCTTGCA-3'<br>5'-CCCTGTTAATCCGTTCGTTTTT-3'
ABCB1	5'- GCTCATCGTTTGTCTACAGTTCGT -3'<br>5'- ACAATGACTCCATCATCGAAACC -3'
β-actin	5'- AGAGCTACGAGCTGCCTGAC -3'<br>5'- AGCACTGTGTTGGCGTACAG -3'

Graphical abstract of the present study

PKM2 inhibition enhanced mitochondrial respiratory activity (1) and upregulated Wnt-FZD expression, thereby driving β-catenin nuclear translocation (2). The increased nuclear PKM2 translocation may form a complex with β-catenin and promote signaling transactivation (3), which consequently augments CSCs formation, self-renewal capacity, chemoresistance, tumorigenicity, and initiates poorly differentiated tumors in patients with head and neck cancer (4).
